# Development of SPR Imaging-Impedance Sensor for Multi-Parametric Living Cell Analysis

**DOI:** 10.3390/s19092067

**Published:** 2019-05-03

**Authors:** Yuhki Yanase, Kyohei Yoshizaki, Kaiken Kimura, Tomoko Kawaguchi, Michihiro Hide, Shigeyasu Uno

**Affiliations:** 1 Department of Dermatology, Graduate School of Biomedical and Health Science, Hiroshima University, 1-2-3 Kasumi, minami-ku, Hiroshima 734-8551, Japan; tomokok@hiroshima-u.ac.jp (T.K.); ed1h-w1de-road@hiroshima-u.ac.jp (M.H.); 2 Department of Electrical and Electronic, Ritsumeikan University, Kusatsu, Shiga 525-8577, Japan; rits.yoshizaki.2018@gmail.com (K.Y.); re0071ep@ed.ritsumei.ac.jp (K.K.)

**Keywords:** surface plasmon resonance (SPR) sensor, SPR imaging (SPRI) sensor, impedance sensor, RBL-2H3 cells, multi-parametric cell analysis

## Abstract

Label-free evaluation and monitoring of living cell conditions or functions by means of chemical and/or physical sensors in a real-time manner are increasingly desired in the field of basic research of cells and clinical diagnosis. In order to perform multi-parametric analysis of living cells on a chip, we here developed a surface plasmon resonance (SPR) imaging (SPRI)-impedance sensor that can detect both refractive index (RI) and impedance changes on a sensor chip with comb-shaped electrodes. We then investigated the potential of the sensor for label-free and real-time analysis of living cell reactions in response to stimuli. We cultured rat basophilic leukemia (RBL)-2H3 cells on the sensor chip, which was a glass slide coated with comb-shaped electrodes, and detected activation of RBL-2H3 cells, such as degranulation and morphological changes, in response to a dinitro-phenol-conjugated human serum albumin (DNP-HSA) antigen. Moreover, impedance analysis revealed that the changes of impedance derived from RBL-2H3 cell activation appeared in the range of 1 kHz–1 MHz. Furthermore, we monitored living cell-derived RI and impedance changes simultaneously on a sensor chip using the SPRI-impedance sensor. Thus, we developed a new technique to monitor both impedance and RI derived from living cells by using a comb-shaped electrode sensor chip. This technique may enable us to clarify complex living cell functions which affect the RI and impedance and apply this to medical applications, such as accurate clinical diagnosis of type I allergy.

## 1. Introduction

Since individual cells are the smallest parts of living organisms, techniques that can monitor conditions and functions of intact living cells in a real-time manner without any labeling are required in the field of life science and clinical diagnosis. Recently, several kinds of chemical and physical biosensors, such as the quartz crystal microbalance (QCM) sensor [1,2], the field-effect transistor (FET) sensor [3], the surface plasmon resonance (SPR) sensor [4], the resonant waveguide grating (RWG) sensor [5], and impedance sensors [6,7,8] have been applied for real-time living cell analysis. These sensors can detect changes of living cell-derived physical or chemical properties on the sensor surface without any labeling in a real-time manner. SPR sensors detect averaged changes of refractive index (RI) in the detection area, or the evanescent field (<500 nm), on the surface of the SPR sensor chip, which is a glass slide coated with thin gold film (50 nm). The RI changes measured by the SPR method reflect various intracellular reactions of cells cultured on the sensor chip, such as the morphology, membrane potential, and density of molecules/proteins. We previously reported that SPR sensors could detect real-time changes of RI in response to the activation of living cells, such as mast cells, keratinocytes, human basophils, and lymphocytes without any labeling, suggesting the potential of the SPR sensor as a tool for cell analysis in basic research and diagnosis for allergy and immunology [9,10,11,12,13,14]. Moreover, we recently developed an SPR imaging (SPRI) sensor in order to visualize the distribution of RI on a sensor chip, a thin gold film (50 nm)-coated glass slide (RI = 1.72). The SPRI sensor could visualize the change of RI distribution induced by treatment with various stimuli, which are inhibitors at single cell levels [15,16,17,18,19]. On the other hand, impedance sensors detect living cell-based impedance changes on an electrode which specifically reflects the attachment and adhesion area of living cells on the electrode at around 10 kHz [20]. We previously reported that impedance sensors (iCELLigence, ACEA Biosciences, Inc., San Diego, CA, USA) can detect changes of the adhesion area of living cells such as RBL-2H3 cells and vascular endothelial cells (HUVECs) at 10 kHz in response to stimuli without any labeling [20,21]. Moreover, we applied the technique for mast cells reaction-based clinical diagnosis of type I allergy [20].

The SPR/SPRI sensors or impedance sensors detect a single parameter of a physical phenomenon on a sensor chip—RI near the plasma membrane of cells, or impedance derived from cells, respectively. Therefore, the information concerning behaviors and functions of living cells monitored by each sensor is limited. Recently, dual biosensing platforms for living cell analysis that can provide complementary information regarding the functions and behaviors of living cells have been reported. Michaelis et al. developed a sensor to detect both impedance and RI changes in living cells at the same time using ECIS-SPR sensors [22]. A technique for simultaneous measurement of RI distribution and cyclic voltammetry, which reflects the condition of living cells, using electrochemical-surface plasmon resonance imaging (EC-SPRI) [23] was proposed by Zhang et al. Although this multi-parametric analysis of cells can provide complementary information regarding the function and behavior of living cells, an SPRI sensor combined with an impedance sensor with a comb-shaped electrode has not yet been developed. The advantages of combining SPRI and impedance methods would include (a) detailed monitoring of cell morphology by SPRI, (b) the fully non-invasive nature of the impedance method, and (c) the potential application to single cell monitoring.

In this study, we developed an SPRI-impedance (SPRI-IMP) sensor and investigated the potential of the sensor for multi-parametric analysis of living cells without any labeling in a real-time manner.

## 2. Materials and Methods

### 2.1. Reagents

Chemicals were obtained from the following sources: Bovine serum albumin (BSA), dinitro-phenol-conjugated human serum albumin (DNP-HSA), DNP-specific rat monoclonal Immunoglobulin E (IgE), and Phalloidin–Tetramethylrhodamine B isothiocyanate (phalloidin-TRITC) from Sigma-Aldrich Japan (Tokyo, Japan). Fetal calf serum (FCS) was from Biowest (Nuaillé, France). Penicillin/streptomycin was from Life Technologies (Carlsbad, CA, USA). FlexiPERM^®^ was from Greiner Bio-One (Kremsmünster, Austria).

### 2.2. Cells Lines

RBL-2H3 cells were cultured in a Roswell Park Memorial Institute (RPMI) medium supplemented with 10% fetal calf serum (FCS), 100 U/mL penicillin, and 100 μg/mL streptomycin. On the day before experiments, cells were harvested using trypsin and then cultured (1 × 10^5^ cells/mL) overnight with anti-DNP IgE (50 ng/mL) in flexiPERM^®^ on an SPR-impedance sensor chip. In general, higher cell density leads to higher signal strength, while an excessive number of cells may suppress the diffusion of DNP-HSA to the lowermost cells. The cell density mentioned above was set as an optimum value.

### 2.3. Instrument for SPR Imaging (SPRI)-Impedance Sensor

**Figure 1:**
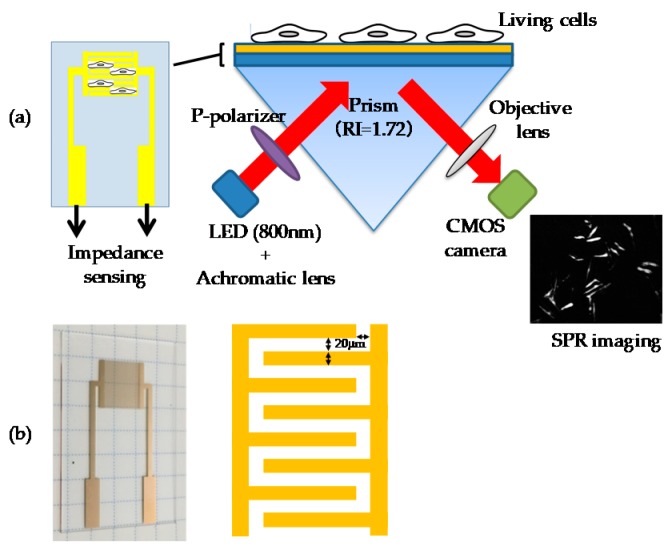
Schematics of surface plasmon resonance imaging-impedance (SPRI-IMP) sensor chip and system for living cell analysis. (**a**) The SPRI-IMP sensor, composed of a light source, P-polarizer, prism, objective lens, and a complementary metal-oxide-semiconductor (CMOS) camera, simultaneously detects living cell-derived refractive index (RI) and impedance changes on a sensor chip. (**b**) SPRI-IMP sensor chip is a glass slide coated with comb-shaped thin gold film in an interdigitated electrode structure with width/spacing of 20 μm.

The SPRI sensor for SPRI-IMP analysis was composed of a light source (800 nm LED, THORLABS, Tokyo, Japan), an achromatic lens (Sigma Koki Co., Ltd., Tokyo, Japan), a P-polarizer (Sigma Koki Co., Ltd.), an equilateral triangle prism (S-LAL10, RI = 1.72), a thermostat and telecentric lens (×0.6, Edmund, Barrington, NJ, USA) and a complementary metal-oxide-semiconductor (CMOS) camera (monochrome-digital CMOS camera, 1280 × 1024 pixel 15 fps, ARTRAY Co., Ltd., Tokyo, Japan), as shown in Figure 1a. SPRI-IMP measurement was performed at around 35 °C controlled with the thermostat (MISUMI Group Inc. Tokyo, Japan). Obtained images and changes of reflected light intensity of indicated areas were analyzed with Image-Pro (Media Cybernetics, Bethesda, MD). The sensor chips (S-LAL10, 40 mm × 26 mm × 1 mm, RI = 1.72) were coated with comb-shaped thin gold film (1.0 nm Cr and a 49 nm gold layer) with finger width/spacing of 20 μm by means of vapor deposition (Osaka Vacuum Industrial Co., Ltd., Osaka, Japan), as shown in Figure 1b. The variation in thickness of thin gold film on a glass slide was confirmed to be within 5% (50 ± 2.5 nm). The quality of the sensor chip stored at room temperature does not decline even after one year. In this article, we focused on the living-cells reactions-associated RI changes and impedance changes. We are planning to apply the technique for several other applications, such as clinical diagnosis of allergy and cancer.

### 2.4. Measurement of Impedance

Impedance was measured using a general-purpose electrochemical analyzer with impedance measurement functionality (ALS/CH electrochemical analyzer model 610DR, BAS). The DC bias was set to zero, and AC voltage amplitude was 5.0 mV. The frequency dependence of impedance magnitude was measured in the frequency range of 10^0^–10^6^ Hz (1 Hz to 1 MHz). The time course of impedance magnitude during the cell stimulation process was recorded once every 10 s at 20 kHz, where the impedance change due to cell stimulation is expected to be observed based on the frequency response measurement results shown in Section 3.3.

### 2.5. Staining of Actin Cytoskeleton in RBL-2H3 Cells

RBL-2H3 cells cultured on the SPRI-impedance sensor chip were fixed with 4% paraformaldehyde 10 min after DNP-HSA stimulation. Following two phosphate-buffered saline (PBS) washes, the cells were treated with PBS containing phalloidin-TRITC for 30 min. Cells on the sensor chip were photographed under a fluorescent microscope (IX83, Olympus, Tokyo, Japan).

### 2.6. Assays of RBL-2H3 Cell Degranulation (Release of β-Hexosaminidase and Histamine)

The degranulation of RBL-2H3 cells cultured on the SPRI-IMP sensor chip was evaluated 15 min after stimulation with the release of β-hexosaminidase, a granule marker, by the hydrolysis of 4-nitrophenyl N-acetyl-β-D-glucosaminide to the chromatophore, which was p-nitrophenol as described previously [9].

## 3. Results

### 3.1. Development of SPRI-IMP Sensor and Sensor Chip

In order to perform multi-parametric detection of the living cell-derived refractive index (RI) and impedance changes on a sensor chip, we first constructed an SPRI-IMP sensor chip, which was composed of a glass slide with a high RI (RI = 1.72) coated with a comb-shaped electrode as shown in Figure 1b. The optical and electrical setup of the SPRI-IMP sensor was prepared as described in the “Materials and Methods” section and shown in Figure 1a. 

### 3.2. Activation of RBL-2H3 Cells Cultured on Comb-Shaped Electrodes

**Figure 2:**
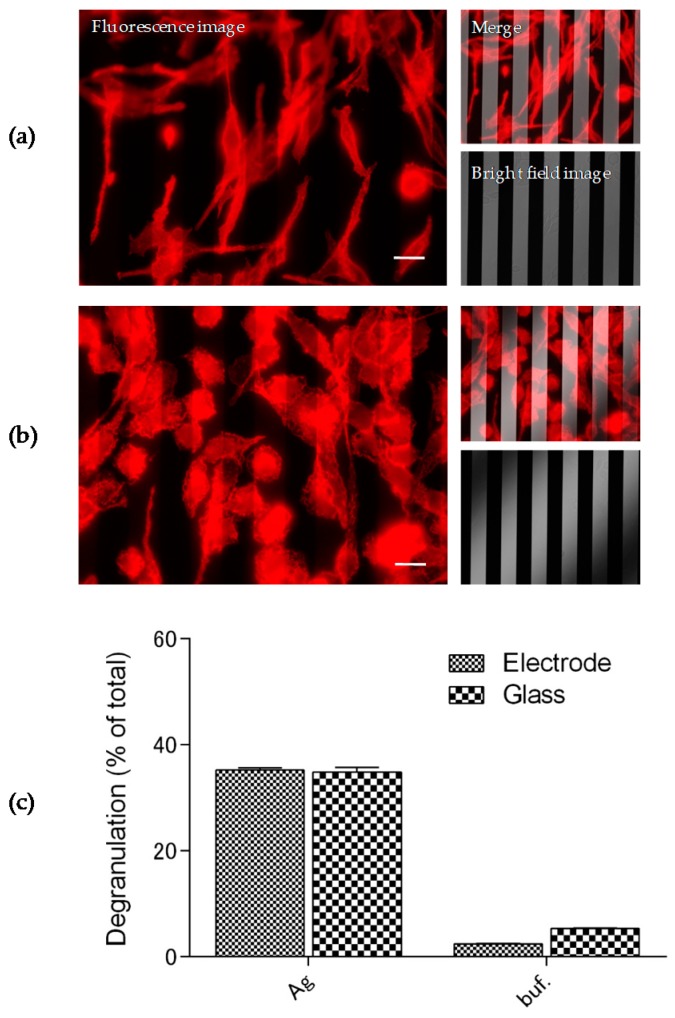
Activation of RBL-2H3 cells in response to stimulation on the comb-shaped electrodes. Change of adhesion area of RBL-2H3 cells before (**a**) and after (**b**) addition of dinitro-phenol-conjugated human serum albumin (DNP-HSA) (Ag) on the comb-shaped electrode was visualized by staining with Phalloidin–Tetramethylrhodamine B isothiocyanate (phalloidin-TRITC). White bars show ca. 20 μm. Half-transparent white bars show 20 μm width and spacing. (**c**) Degranulation of RBL-2H3 cells in response to an antigen on the SPR-IMP sensor chip. Degranulation of RBL-2H3 cells was evaluated with the release of β-hexosaminidase, a granule marker, by the hydrolysis of 4-nitropheny-N-acetyl-β-glucosaminide to the chromatophore, p-nitrophenol. The data is representative of three independent experiments, each performed in triplicate.

We first investigated if comb-shaped electrodes affect the activation, morphological change, and degranulation of rat basophilic leukemia (RBL)-2H3 cells, widely used as a mast cell model, in response to a DNP-HSA antigen on the sensor chip. In order to activate RBL-2H3 cells by a DNP-HSA antigen, the cells were cultured in the presence of anti-DNP IgE antibodies (50 ng/mL) overnight before stimulation with DNP-HSA. Figure 2a,b shows RBL-2H3 cells before (a) and after (b) stimulation stained with phalloidin-TRITC, which specifically binds to actin filaments in cells, on the SPRI-IMP sensor chip. When RBL-2H3 cells were stimulated with DNP-HSA (50 ng/mL), morphological changes, such as ruffling and spreading, were observed even on the sensor chip with the comb-shaped electrode, as seen in Figure 2b. Moreover, degranulation, evaluated by the release of β-hexosaminidase, from RBL-2H3 cells in response to DNP-HSA (50 ng/mL) was also detected even on the comb-shaped electrodes, as shown in Figure 2c. These results suggest that comb-shaped electrodes do not affect the adhesion of RBL-2H3 cells to the surface of the sensor chip or activation of the cells, such as morphological changes and degranulation, in response to the antigen.

### 3.3. Frequency Characteristics of Impedance of RBL-2H3 with or without Stimulation

**Figure 3:**
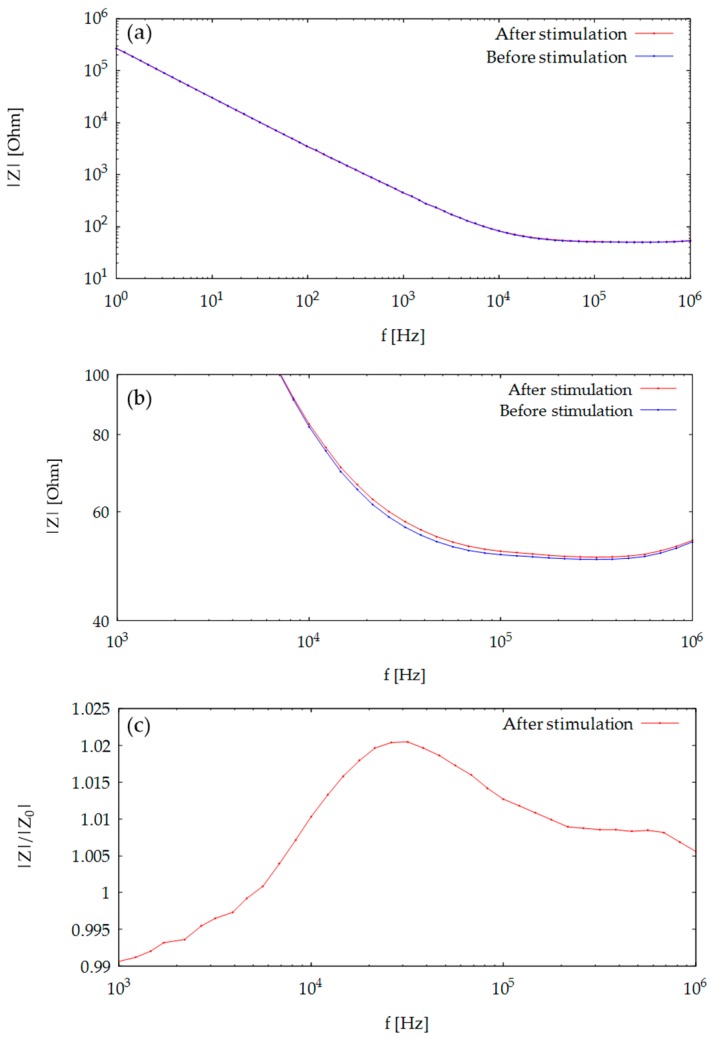
Frequency characteristics of impedance of RBL-2H3 with or without stimulation. (**a**) Impedance magnitude as a function of frequency. (**b**) Magnified view of the result shown in (**a**) around the corner of |Z| change. (**c**) Impedance magnitude after stimulation normalized by that before stimulation, showing impedance increase due to cell activation.

**Figure A1:**
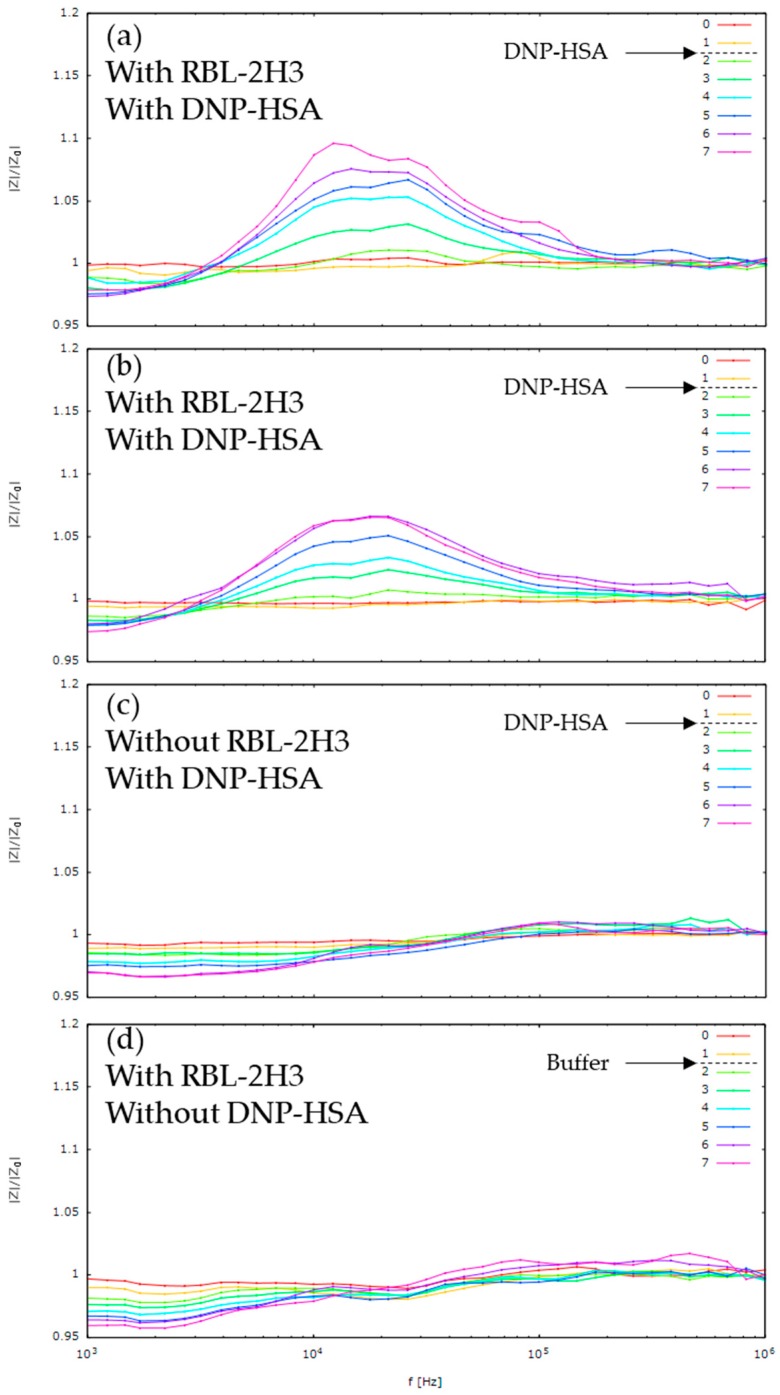
Repeated and control experiments of impedance changes due to cell stimulation. Initially, cells are in buffer solution, and impedance measurements were done every 4 min, numbered from 0 to 7 in sequential order. Buffer or DNP-HSA are injected after measurement 1 is taken. Differences in experimental conditions from Figure 3 are the sensor Au thickness (100 nm) and incubator used (YAMATO IC402). (**a**) and (**b**) were taken with RBL-2H3 and DNP-HSA, (**c**) experiment without cells on sensor surface, (**d**) experiment without DNP-HSA while cells are on the sensor.

We then measured the frequency dependence of impedance with RBL-2H3 cells cultured on the SPRI-IMP sensor chip before and after stimulation with DNP-HSA. Figure 3a shows impedance magnitude as a function of frequency before (blue line) and after (red line) the stimulation, and Figure 3b shows a zoom-up in the frequency range of 1 kHz–1 MHz. As can be seen in the figure, the impedance magnitude was increased after stimulation due to the increase of cell coverage on the electrode surface. Figure 3c shows the impedance magnitude at each frequency point after stimulation (Z) divided by that before stimulation (Z_0_), which exhibits the ratio of the impedance change after stimulation. Repeated measurements with similar experimental conditions were made (Figure A1a,b in the Appendix A), and control experiments without cells or without DNP-HSA were also performed, see Appendix A, Figure A1c,d to confirm that this increase was due to cell stimulation. Thus, the sensor could detect morphological changes of RBL-2H3 cells on comb-shaped electrodes. As can be observed in Figure 3c, the maximum impedance increase was around 30 kHz. By repeating the same experiments with different batches of cells/sensors, we observed that the maximum increase appeared around 10 kHz to 30 kHz, see Appendix A, Figure A1a,b. In the following time-dependent impedance measurement, we used 20 kHz as the fixed frequency of monitoring.

### 3.4. Simultaneous Monitoring of Living Cell-Derived RI and Impedance on an SPRI-Impedance Chip

**Figure 4:**
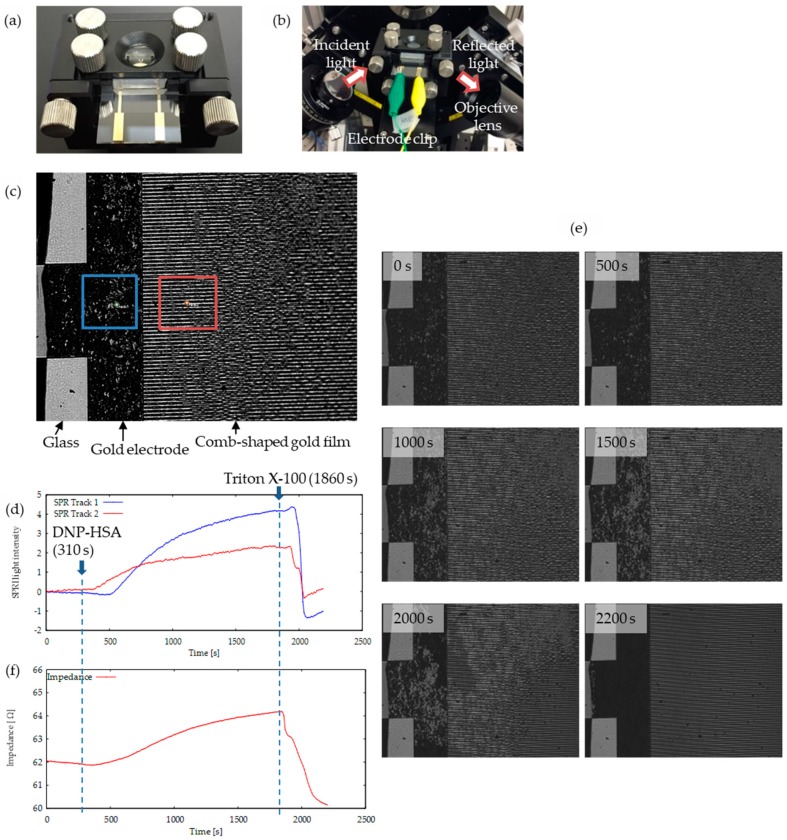
Simultaneous monitoring of living cell-derived RI and impedance on an SPRI-IMP chip. (**a**) SPRI- IMP sensor chip holder. (**b**) Set up of SPRI-IMP sensor. (**c**) SPR image of SPRI-IMP sensor chip on which RBL-2H3 cells are cultured. (**d**) Reflected light intensity changes of RBL-2H3 cells on gold film area (blue line) and comb-shaped area (red line) in response to DNP-HSA. (**e**) Snapshots of SPRI at the indicated times. (**f**) Simultaneous monitoring of RI and impedance based on RBL-2H3 cell reactions in response to DNP-HSA.

**Figure A2:**
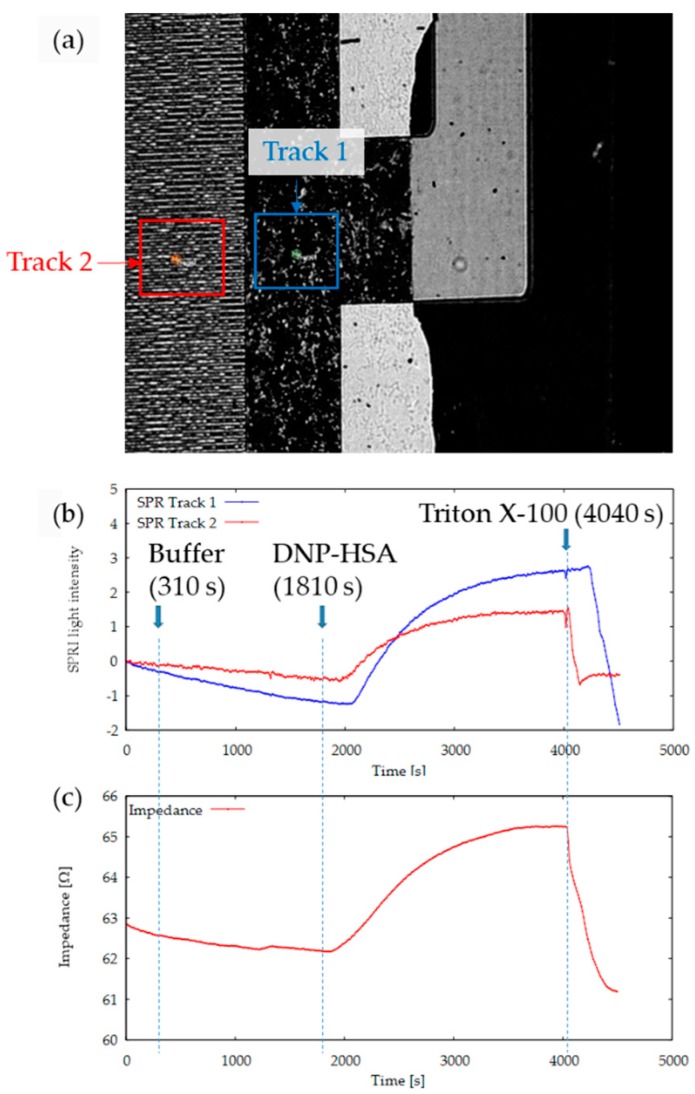
Simultaneous monitoring of RI and impedance changes of RBL-2H3 cells cultured on a SPRI-IMP chip in the absence or presence of DNP-HSA. RI and impedance were not increased after addition of buffer without DNP-HSA. On the other hand, both RI and impedance were increased after stimulation with DNP-HSA, and decreased after addition of Triton X-100. (**a**) SPR image of SPRI-IMP sensor chip on which RBL-2H3 cells are cultured. (**b**) Reflected light intensity changes of RBL-2H3 cells, (**c**) Simultaneous monitoring of RI and impedance.

Finally, we performed simultaneous multi-parametric analysis of RBL-2H3 cells cultured on the SPRI-IMP sensor chip in response to the DNP-HSA antigen (50 ng/mL). The SPRI-IMP sensor chip with an RBL-2H3 cell culture was set in a prism holder, as shown in Figure 4a. SPRI-IMP monitoring was then performed as shown in Figure 4b. The angle of the incident light was fixed at the resonance angle of buffer solution (around 50°). This was done to ensure that the light reflection from the area with the gold electrode would be weak and shown to be dark in the CMOS camera image, while the other area without the gold electrode would be bright. Additionally, if there were RBL-2H3 cells adhered on the gold electrode, those cells would change the RI near the electrode surface and would result in bright spots. Such an image is shown in Figure 4c, where the comb-shaped gold electrode, glass substrate, and bright spots due to RBL-2H3 cells are clearly seen. Figure 4d indicates the time course of reflected light intensity, which is related with RI changes on the gold surface, taken at different areas indicated by the red and blue square in Figure 4c. Initially, cells were in buffer solution, and DNP-HSA was added at 310 s to initiate cell activation. Then, Triton X-100 was added at 1860 s, which dissolved the lipid bilayer of the plasma membrane and left adhesion molecules on the sensor chip. As can be seen in the figure, the RI of individual RBL-2H3 cells in response to the DNP-HSA antigen (50 ng/mL) increased both on gold film (the area of the blue square in Figure 4c), and the comb-shaped electrode (the area of the red square in Figure 4c). The snapshots of SPRI at some selected times are shown in Figure 4e (the actual movie is provided in the Supplementary Materials). In addition to the SPRI measurement, impedance monitoring was simultaneously performed and changes in the impedance value were observed in synchronization with the SPR measurement as shown in Figure 4f. The impedance value at 20 kHz reflects the solution resistance due to ionic movement between the interdigitated electrodes, and, therefore, it was a measure of cell coverage increase due to the activation of RBL-2H3 cells. As can be seen in the figure, the impedance increased as the cell coverage of the electrodes increased. Furthermore, this agrees well with the changes in SPRI intensity or RI changes. In order to confirm if the addition of the buffer itself increased RI and the impedance of RBL-2H3 cells, we then performed a control experiment, see Appendix A, Figure A2. Although an increase of RI and impedance were not observed for 30 min in the absence of specific stimulation (DNP-HSA), both the RI and impedance of RBL-2H3 cells immediately increased when DNP-HSA was added. Thus, we could successfully perform the simultaneous monitoring of RI and impedance changes that were derived from reactions of RBL-2H3 cells in response to specific stimulation. Note that a slight delay of SPRI intensity response to the addition of DNP-HSA and Triton X-100 compared to IMP response might be due to the difference in sensing area. In IMP measurement, the output will be an averaged response on a whole comb-shaped electrode area, while in SPRI, the signal is taken in a localized area indicated in Figure 4c. As the molecules were added by a pipette from the top of the buffer, there was a slight time delay in the different areas of the sensor. Such a delay, as shown in Figure 4d, can easily be minimized by reducing the size of the comb-shaped electrode area.

## 4. Discussion

In this study, we established a technique to perform multi-parametric living cell analysis using an SPRI sensor integrated with an impedance sensor—an SPR-IMP sensor. Using the technique, we successfully monitored RI and impedance, which reflect the condition of RBL-2H3 cells cultured on a sensor chip without any labeling. SPRI sensors can visualize and detect RI changes in and out of a plasma membrane, which reflect the distribution of living cell-derived lipids, proteins, and ions in the SPR detection area (<500 nm). On the other hand, the impedance sensor used here (the frequency was 20 kHz) mainly detects living cell properties in the electrical insulation on an electrode that reflects the coverage area and morphology (and potentially membrane microstructure), which cannot be observed under an optical microscope, of attached cells on the surface of the electrode. However, single parametric analysis by means of SPR or impedance is insufficient for analysis of extremely complex functions of living cells, suggesting the necessity of multi-parametric cell analysis based on impedance and RI using an SPRI-IMP sensor.

SPRI and impedance sensors have been widely applied for living cell analysis. As for SPRI sensors, we applied the SPRI sensor for clinical diagnosis of type I allergy based on the reactions of mast cells and basophil on the sensor chip in response to specific antigens [16,18]. Peng et al. monitored epidermal growth factor (EGF) receptor-induced cell responses using an SPRI system [24]. Moreover, the application of an SPR-imaging sensor for monitoring the translocation of protein kinase C in PC12 cells has been reported by Shinohara et al [25]. Furthermore, Peterson et al. reported a technique to visualize the interactions of the cell-extracellular matrix using an SPRI sensor [26]. Meanwhile, the impedance method has been applied for non-invasive living cell monitoring for oocytes [27], bladder cancer cells [28], oral cancer cells [29], platelets [30], circulating tumor cells [31], red blood cells [32], and other various kinds of cells [33,34]. Therefore, the system we developed here could potentially be applied for multi-parametric living cell analysis of various kinds of cells. Incorporating SPRI and impedance methods would thus provide a wide variety of applications.

As we described above, an SPR/SPRI sensor can detect RI changes in a detection area on gold film (<500 nm) that is much smaller than cell height (>1 μm). In order to extend the detection area of SPR/SPRI sensors, long-range SPR (LRSPR) sensors for living cells have been reported [35,36]. Since LRSPR sensors enhance the detection depth by more than 1 μm, they are capable of detecting a RI change of living cells deeper than conventional SPR sensors. Therefore, the analysis of living cells using LRSPR sensors enables us to clarify the detailed information on RI change mechanisms of living cells. On the other hand, the resolution of an SPR imaging sensor is around 1 μm, which is worse than that of an optical microscope. In order to improve the resolution of an SPR image, a high-resolution SPRI technique has been reported by Peterson et al [37]. In addition, although we have demonstrated impedance measurements up to 1 MHz frequency in this work, measurements using higher frequencies (up to several tens of MHz) have been used to observe intra-cellular information, such as cell membrane and cytoplasm [33,34]. Such high-frequency measurement in cooperation with SPRI measurement will provide more detailed monitoring of cell physiology. Additionally, simultaneous SPRI-IMP measurement will be possible even with small electrodes up to a single cell level. Such experimental setup will show the physiological responses of individual cells, which will be necessary in many applications such as zygote monitoring.

## 5. Conclusions

In this study, we developed a new technique to detect both RI and impedance changes of living cells cultured on a sensor chip. Further analysis of living cells by the sensor would enable us to obtain more detailed information on living cell conditions and functions that cannot be clarified by conventional optical and physicochemical biosensors.

## Supplementary Materials

The following movie are available online at https://www.mdpi.com/1424-8220/19/9/2067/s1. Real-time monitoring of RI distribution on the SPRI-impedance sensor chip. Refracted light intensity of individual cells is increased in response to DNP-HSA and then decreased by treatment with Triton X.

## Appendix A

The following figures show data taken for control experiments and reproducibility check.
